# Low-metastatic melanoma cells acquire enhanced metastatic capability via exosomal transfer of miR-199a-1-5p from highly metastatic melanoma cells

**DOI:** 10.1038/s41420-022-00993-8

**Published:** 2022-04-09

**Authors:** Qiting Zhao, Hao Chen, Xiaoshuang Li, Bin Zeng, Zhiwei Sun, Doudou Liu, Yuting Chen, Yuhan Zhang, H. Rosie Xing, Jianyu Wang

**Affiliations:** 1 State Key Laboratory of Ultrasound in Medicine and Engineering, College of Biomedical Engineering, Chongqing Medical University, Chongqing, 400016 China; 2 Chongqing Key Laboratory of Biomedical Engineering, Chongqing Medical University, Chongqing, 400016 China; 3 Institute of Life Sciences, Chongqing Medical University, Chongqing, China

**Keywords:** Melanoma, Metastasis

## Abstract

The mean survival of metastatic melanoma is less than 1 year. While the high mortality rate is associated with the efficient metastatic colonization of the involved organs, the underlying mechanisms remain elusive. The role of exosomes in facilitating the interactions between cancer cells and the metastatic microenvironment has received increasing attention. Previous studies on the role of exosomes in metastasis have been heavily focused on cancer cell-derived exosomes in modulating the functions of stromal cells. Whether the extravasated neighboring cancer cells at the distant organ can alter the metastatic properties of one another, a new mechanism of metastatic colonization, has not been demonstrated prior to this report. In this study, a paired M4 melanoma derivative cell lines, i.e., M14-OL and POL, that we established and characterized were employed. They exhibit high (POL cells) and low (OL cells) metastatic colonization efficiency in vivo, respectively. We show that exosomal crosstalk between metastatic cancer cells is a new mechanism that underlies cancer metastasis and heterogeneity. Low metastatic melanoma cells (OL) can acquire the “metastatic power” from highly metastatic melanoma cells (POL). POL achieves this goal by utilizing its exosomes to deliver functional miRNAs, such as miR-199a-1-5p, to the targeted OL cell which in turn inactivates cell cycle inhibitor CDKN1B and augments metastatic colonization.

## Introduction

Cutaneous melanoma is the most common form of skin cancer [1, 2] and the incidence is on the rising worldwide [3, 4]. The mean survival of metastatic melanoma is less than 1 year [5]. Although the targeted and immunotherapies have brought hopes to locally invasive and metastatic melanoma patients, the development of resistance to the new line of therapies remains the bottleneck for melanoma management. While the high mortality rate of metastatic melanoma is related to the efficient metastatic colonization of the involved organs, notably the brain and the lung, the underlying mechanisms remain elusive.

The efficiency of proliferative colonization of the extravasated cancer cells at the distant organ is rate-limiting for the development of clinically detectable metastases [6–8]. At the stage of colonization, a new microenvironment for tumor growth must be formed. A plethora of factors and mechanisms are involved in this process. Interactions between cancer cells and their tissue microenvironment, achieved via direct cell–cell interactions, or via indirect paracrine interactions in metastatic dissemination have received increasing appreciation [9].

The role of a new class of mediators, the exosomes, in the establishment of metastatic niche or microenvironment, is at the forefront of cancer research. Exosomes are membranous micro-vesicles, sized 40–100 nm, that play an important part in cell–cell communication [10]. The exosomal crosstalk involves the transfer of “messengers” including miRNA, mRNA and proteins between donor cells and the neighboring recipient cells [11]. Exosomes may modulate the biological functions, or cellular fate or properties of the target cells depending on the content of exosomes, and hence may influence the course of cancer initiation, progression or development of resistance to therapies [12, 13]. Previous studies on the role of exosomes in metastasis have been heavily focused on cancer cell-derived exosomes in modulating the functions of stromal cells (endothelial cells, fibroblasts, and immune cells) at the involved organ to support the clonogenic proliferation of the metastatic cells [14]. There is increasing evidence demonstrating the ability of stromal cell-derived exosomes in modifying of the metastatic behavior of cancer cells [15, 16]. Few studies show that cancer stem cell (CSC)-derived exosomal miRNA can enhance EMT (epithelial to mesenchymal transition) of non-CSCs [17]. Whether the extravasated neighboring cancer cells at the distant organ can alter the metastatic properties of one another, a new mechanism of metastatic colonization, has not been demonstrated prior to this report.

In this study, a paired M4 melanoma derivative cell lines, i.e., M14-OL and POL, that we established and characterized were employed [18]. They exhibit high (POL cells) and low (OL cells) metastatic colonization efficiency in vivo, respectively. We show that exosomal crosstalk between metastatic cancer cells is a new mechanism that underlies cancer metastasis and heterogeneity. Low metastatic melanoma cells (OL) can acquire the “metastatic power” from highly metastatic melanoma cells (POL). POL achieves this goal by utilizing its exosomes to deliver functional miRNAs, such as miR-199a-1–5p, to the targeted OL cell which in turn inactivates cell cycle inhibitor CDKN1B and augments metastatic colonization.

## Results

### Exosomes secreted by high-metastatic POL cells can promote the invasiveness of the low-metastatic OL cells

**Fig. 1:**
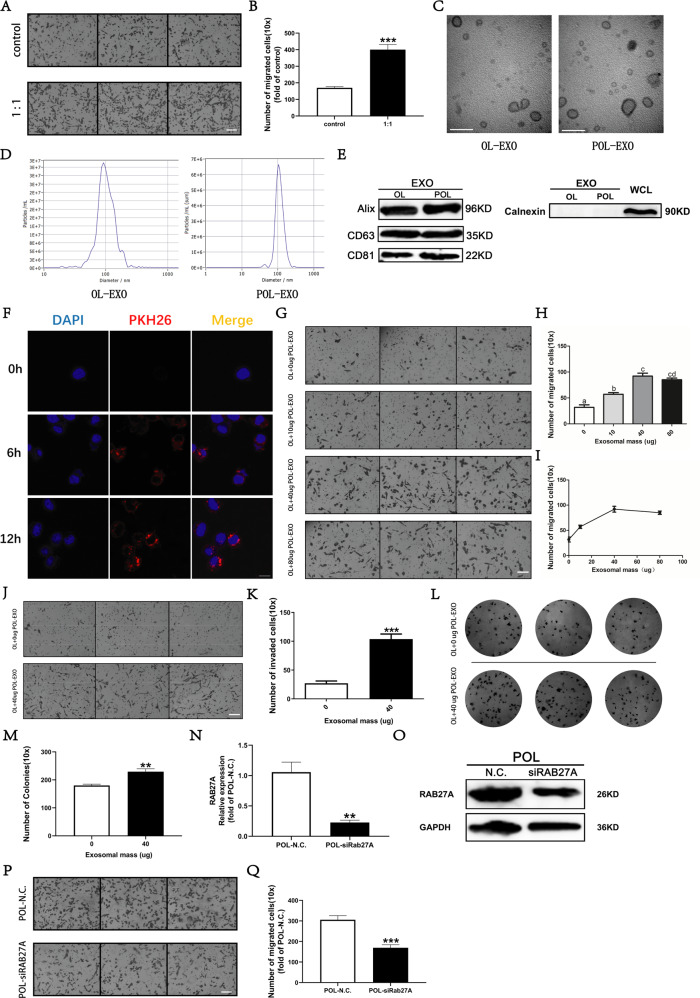
POL regulates the progression of OL to POL by secreting exosomes. **A** The change of migration ability of OL cells after co-culture with POL cells, bar = 60 μm. **B** Quantitative assessment of the change of migration ability of OL cells after co-culture with POL cells, ***P < 0.001. TEM analysis **(**bar = 100 nm, **C**), NTA analysis (**D**) and WB analysis of protein markers (**E**) of exosomes secreted by OL and POL cells. **F** Imaging analysis of the time course of POL-exo uptake by OL cells, bar = 10 μm. Visualization (**G**, bar = 60 μm) and quantitative analysis (**H**) of the effect of exosomes secreted by POL (POL-exo) on the migration ability of OL. **I** The dose effect of POL-exo on migration ability of OL. Visualization (**J**, bar = 60 μm) and quantitative analysis (**K**, ***P < 0.001) of the effect of exosomes secreted by POL (POL-exo) on the invasiveness of OL cells. Visualization (**L**) and quantitative analysis (**M**, **P < 0.01) of the effect of exosomes secreted by POL (POL-exo) on the clonogenic proliferation of OL cells. **N** RAB27A mRNA expression (**N**, **P < 0.01) and protein expression (**O**) in POL-N.C.and POL-siRAB27A. Visualization (**P**, bar = 60 μm) and quantitative analysis (**Q**, ***P < 0.001) of the effect of siRAB7A on the invasiveness of OL cells.

To set up the co-culture experiment, OL and POL cells, placed in the lower and upper chamber of the Transwell, respectively were separated by a 0.4 μm por-sized membrane. After co-culturing for 48 h, we found that co-cultured OL cells exhibited significantly enhanced migration and invasion (Fig. 1A, B). This observation indicates that POL cells may affect the invasive properties of the OL cells by paracrine mechanisms. We focused mechanistic exploration on POL secreted exosomes.

Exosomes were isolated and purified from the culture supernatants of POL cells. The quality and the purity of the obtained exosomes were analyzed by transmission electron microscopy (TEM) for their vesicle morphology (Fig. 1C); by nanoparticle tracking analysis (NTA) for their size range (Fig. 1D); and by Western blot for the expression of exosomal marker proteins Alix, CD63, CD81 and Calnexin (Fig. 1E, Fig. S1A–D).

To prove that POL-derived exosomes (POL-exo) could enter OL cells, POL-exo were labeled with the PKH26 and incubated with OL cells. Confocal imaging analysis showed the time course of the PKH-stained POL-exo uptake by OL cells which peaked at 12 h after the addition of the POL-exo. The majority of the POL-exo were in the cytosol (Fig. 1F) of OL cells.

Next, we evaluated the effects of POL-exo on the metastatic function of OL cells in vitro. In the migration assay, OL cells were incubated with escalating doses of 0, 20, 40, or 80 μg POL-exo, respectively. We observed dose-dependent significant increases in the number of migratory OL cells (Fig. 1G–I). The most pronounced increase was at the addition of 40 μg of POL-exo. We thus chose 40 μg for additional functional testing. OL cells incubated with 40 μg of POL-exo displayed increased invasiveness (Fig. 1J, K) and colony-forming abilities (Fig. 1L, M) compared to the control cells incubated with PBS.

To confirm that the effects we observed with the addition of POL-exo were indeed caused by the POL secreted exosomes, exosome secretion function of POL cells was abrogated by RAB27A siRNA silencing [19, 20]. Effective inhibition of RAB27A expression was confirmed by RT-qPCR (Fig. 1N) and WB (Fig. 1O, Fig. S1E, F), respectively. Next, we co-cultured OL cells with POL-N.C cells or POL-siRAB27A cells, respectively. As expected, inhibition of RAB27A largely prevented the increase of the number of migratory OL cells seen after co-culturing with POL-N.C cells (Fig. 1A, B, P, Q).

Collectively, these observations demonstrate that POL-exo can augment the metastatic function of OL cells in vitro. Since increasing evidence begins to elucidate the importance of exosomal miRNAs in cancer development and progression, we focused our mechanistic investigation on exosomal miRNAs.

### POL-exo and OL-exo miRNA Profiling and prioritization of miR-199a-1-5p

**Fig. 2:**
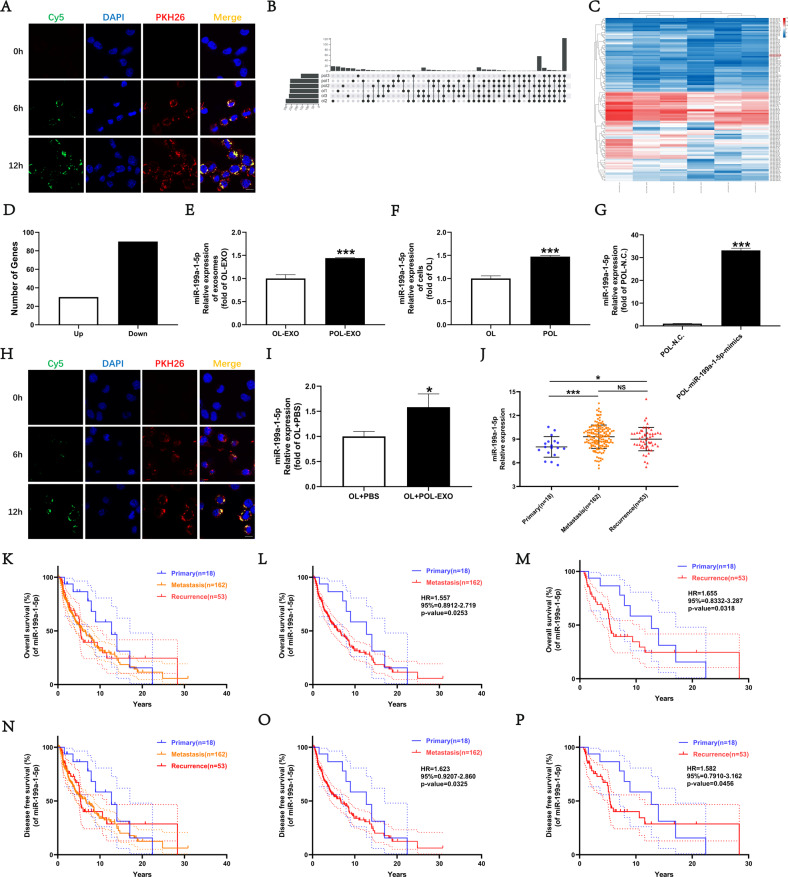
The expression levels of miR-199a-1-5p are significantly increased in POL and POL-EXO, and is enhanced in the OL upon uptaking of POL-exo. **A** Imaging analysis of the time course of OL uptake of exosomes secreted by POL-N.C., bar = 10 μm. Wien diagram (**B**) and Heat map analysis (**C**) of miRNAs secreted by OL and POL cells. **D** Differentially expressed miRNAs between POL-exo and OL-exo. miR-199a-1-5p expression in OL-exo and POL-exo (**E**) and in OL and POL cells (**F**) evaluated by RT-qPCR. ***P < 0.001. **G** miR-199a-1-5p expression in POL-N.C. and POL-miR-199a-1-5p-mimics evaluated by RT-qPCR. ***P < 0.001. **H** Imaging analysis of the time course of OL uptake of exosomes secreted by POL-miR-199a-1-5p-mimics cells, bar = 10 μm. Wien diagram. **I** miR-199a-1-5p expression in OL + PBS and OL + POL-miR-199a-1-5p-mimics-Exo evaluated by RT-qPCR. *P < 0.05. **J** TCGA analysis of miR-199a-1-5p gene relative expression in melanoma. **K**–**P** The Kaplan-plot of miR-199a-1-5p gene in patients with melanoma.

We first set out to determine whether miRNAs in POL-exo can be taken up by OL cells. First, POL cells were transfected with Cy5-labled miRNA N.C mimics. Exosomes were isolated from Cy5-mimics transfected POL cells and labeled with PKH26. Afterwards, PKH26-labeled POL-exo were incubated with OL cells. As confocal imaging is shown in Fig. 2A, within 12 h observation time, PKH-labeled POL-exo entered OL cells. In addition, Cy5-labeled miRNA mimic N.C were found inside OL cells. Co-localization of Cy5-miRNA mimics with PKH26-POL-exo indicates that miRNAs within the POL-exo can be transferred to OL cells.

Next, we conducted miRNA expression profiling of POL-exo and OL-exo by miRNA sequencing. 381 miRNAs exhibited differential expression between the POL-exo and OL-exo, among which 124 were present in all samples (Fig. 2B).

Since we are searching for exosomal miRNAs that promote cancer metastasis, we focused our analysis, prioritization and validation on the 34 miRNAs that exhibited elevated expression in POL-exo (Fig. 2C, D). We used RT-qPCR for validation in OL and POL cells. Finally, we chose miR-199a-1-5p which was most consistent in RT-qPCR for mechanistic investigation.

We first elevated miR-199a-1-5p expression was observed in POL cells and POL-exo compared with OL and OL-exo by RT-qPCR, respectively (Fig. 2E, F). We next confirmed that miR-199a-1-5p can be transferred from POL cells to OL cells by POL-exo. We transfected POL cells with Cy5-labeled miR-195a-1-5p mimics, and confirmed its increased expression by RT-qPCR (Fig. 2G). Exosomes were extracted and labeled with PKH26 and incubated with OL cells. Shown in Fig. 2H, within 12 h observation time, PKH-labeled POL-exo entered OL cells and accumulated mostly in the cytosol. In addition, Cy5-labeled miRNA-199a-1-5p mimics were found inside OL cells. Co-localization of Cy5- miRNA-199a-1-5p mimics with PKH26-POL-exo indicates that miRNA-199a-1-5p can be transferred from POL to OL cells via exosomes. The increase in miR-199a-1-5p expression in OL after 48 h incubation with POL-miR-195a-1-5p mimics-exo was confirmed by RT-qPCR (Fig. 2I). This set of observations show that POL cells can increase the expression of miR-199a-1-5p in OL cells by exosomal transfer of this miRNA to the target cells.

Both oncogenic and tumor suppressor activities of miR-199a-1-5p were reported in melanoma [21–23]. Since miR-199a-1-5p is elevated in POL cells and POL-exo and its high expression is associated with augmented invasiveness, it appears oncogenic in our cellular models. To establish the clinical relevance of miR-199a-1-5p, we analyzed the TCGA datasets by ACLBI database. miR-199a-1-5p expression in metastatic and recurrence tumor samples appeared significantly higher than that in the primary tumor samples. However, no significant difference in miR-199a-1-5p expression was observed between the metastatic and recurrence tumor samples (Fig. 2J). Overall survival (OS) and disease-free survival (DFS) were analyzed. High miR-199a-1-5p expression in metastatic and recurrence patients was significantly associated with poor OS and DFS (Fig. 2K–P). This set of analyses indicates that miR-199a-1-5p is a poor prognostic marker for melanoma and appears oncogenic and pro-metastatic.

### miR-199a-1-5p promotes the metastatic colonization ability of OL cells in vitro and in vivo

**Fig. 3:**
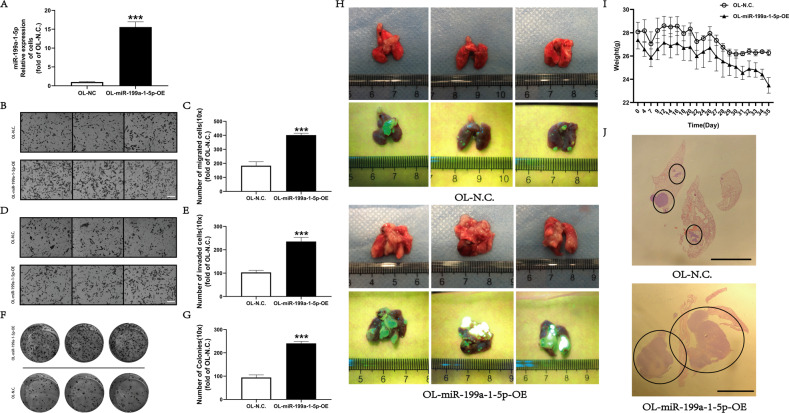
Overexpression of miR-199a-1-5p expression effectively enhanced the metastatic colonization ability of OL in vitro and in vivo. **A** miR-199a-1-5p expression in OL-N.C. and OL-miR-199a-1-5p-OE evaluated by RT-qPCR. ***P < 0.001. Visualization (**B**, bar = 60 μm) and quantitative analysis of (**C**, ***P < 0.001**)** the migration ability of OL-N.C. and OL-miR-199a-1-5p-OE cells. Visualization (**D**, bar = 60 μm) and quantitative analysis of (**E**, ***P < 0.001) of the invasive ability of OL-N.C. and OL-miR-199a-1-5p-OE Cells. Visualization (**F**) and quantitative analysis (**G**, ***P < 0.001) of the clonogenic ability of OL-N.C. and OL-miR-199a-1-5p-OE cells. **H** Analysis of the effect of miR-199a-1-5p overexpression on metastatic colonization in vivo. **I** The weight changes of mouse injected with OL-N.C. and OL-miR-199a-1-5p-OE cells. **J** H&E staining of metastatic nodules in the lungs, bar = 5 mm.

To evaluate the effect of miR-199a-1-5p on melanoma metastasis, stable overexpression of miR-199a-1-5p in OL cells was achieved via lentiviral infection. miR-199a-1-5p overexpression was confirmed by RT-qPCR in OL-miR-199a-1-5p-OE cells (Fig. 3A). Compared with OL-N.C control cells, miR-199a-1-5p overexpression led to enhanced in vitro migratory (Fig. 3B, C) and invasive ability (Fig. 3D, E), as well as clonogenic colonization capability (Fig. 3F, G) in OL-miR-199a-1-5p-OE cells.

To study the effect of miR-199a-1-5p overexpression on OL metastasis in vivo, 10^5^ OL-N.C and OL-miR-199a-1-5p cells were injected into NOD/SCID mice via the tail vein. Weight changes were monitored (Fig. 3I) and all mice were sacrificed at day 35 after tumor cell injection. As shown in Fig. 3H, injected OL-miR-199a-1-5p cells produced significantly more macroscopic lung foci than the OL-N.C cells. H&E analysis was conducted and more large metastatic foci were present in the lungs that received OL-miR-199a-1-5p cells (Fig. 3J). These observations show that miR-199a-1-5p can enhance the metastatic colonization efficiency of OL cells. So far, we have demonstrated that miR-199a-1-5p, transferred from POL-derived exosomes to OL cells, can effectively augment the clonogenic colonization capability of OL cells to give arise to more exrensive macroscopic metastases.

### The pro-metastatic activity of miR-199a-1-5p is achieved through targeting the cell cycle inhibitor CDKN1B

**Fig. 4:**
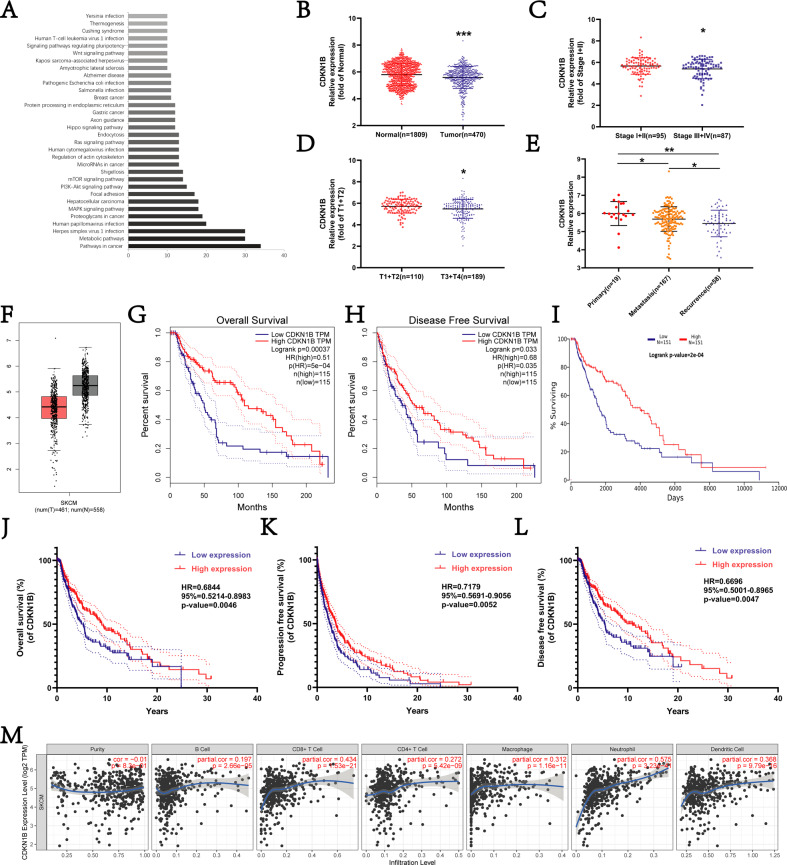
Bioinformatic analysis of CDKN1B gene in human melanoma. **A** KEGG pathway analysis of the target genes of miR-199a-1-5p. **B**–**F** CDKN1B gene relative expression in melanoma. **G**–**L** The Kaplan-plot of CDKN1B gene in patients with melanoma. **M** The association of CDKN1B expression and immune response.

To investigate the molecular mechanisms that mediate the oncogenic activities of miR-199a-1-5p, we used Target Scan Human to determine the predicted gene targets of miR-199a-1-5p. KEGG analysis of the predicted target genes of miR-199a-1-5p prioritized “Pathways in cancer” as the enriched molecular pathway (Fig. 4A). Literature analysis of the target genes in the enriched pathway identified CDKNIB, a well-characterized cell cycle inhibitor and a gene target for the oncogenic activity of miR-199a-1-5p in sarcoma [24].

Since the role of miR-199a-1-5p/CDKN1B axis in melanoma has not been reported, the clinical relevance of CDKN1B was evaluated in human melanoma using the clinical TCGA database, ACLBI database, GEPIA2 database and OncoLnc database.

The expression of CDKN1B was significantly higher in cancer tissues than in the para-cancerous normal tissues (Fig. 4B, F). However, with the progression of melanoma, the expression of CDKN1B was significantly lower in the melanoma (Fig. 4C–E). Survival analysis shows that high-level expression of CDKN1B conferred better OS (Fig. 4G, I, J), progression-free survival (PFS) (Fig. 4K), and DFS (Fig. 4H, L). TIMER database analysis revealed a positive correlation between CDKN1B expression and the number of infiltrating immune cells (B cell, CD8+ cell, CD4+ cell, macrophage, and dendritic cell) (Fig. 4M). These findings collectively indicate that decreased CDKN1B expression is a prognostic marker for poor prognosis (disease progression, recurrence, and survival) for human melanoma.

**Fig. 5:**
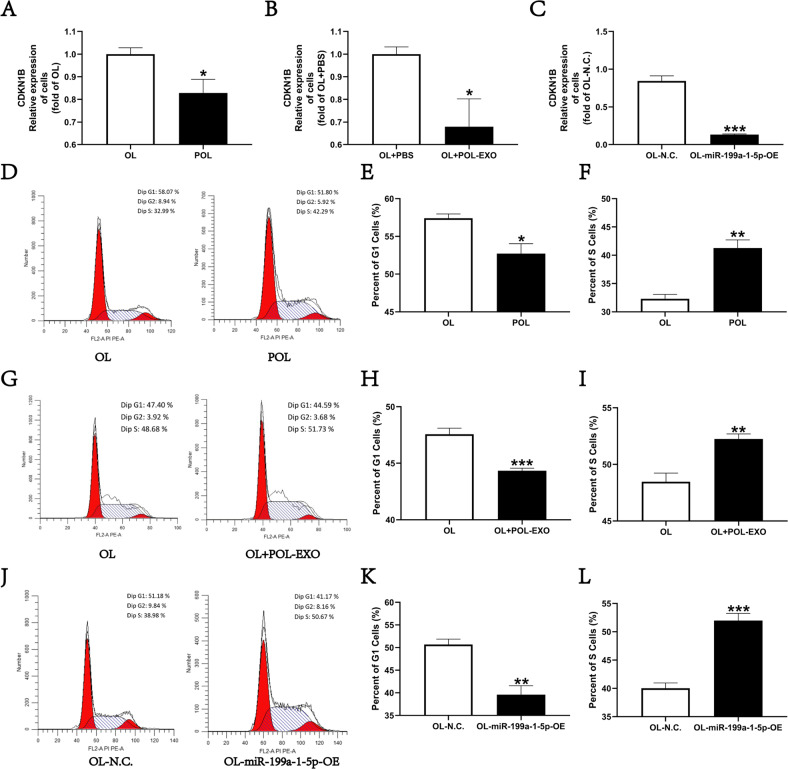
miR-199a-1-5p reduces the expression level of CDKN1B and promotes the cell-cycle transition. CDKN1B gene expression in OL and POL cells (**A**, *P < 0.05), in OL + PBS and OL + POL-exo (**B**, *P < 0.05) and in OL-N.C. and POL-miR-199a-1-5p-OE cells (**C**, ***P < 0.001) evaluated by RT-qPCR. **D** Analysis of changes in cell cycle transition between OL and POL cells. The number of G1 phase (**E**, *P < 0.05), S phase (**F**, **P < 0.01) cells in OL and POL cells. **G** Analysis of changes in cell cycle transition between OL and OL + POL-Exo. The number of G1 phase (**H**, ***P < 0.001) and S phase (**I**, **P < 0.01) cells in OL and OL + POL-Exo. **J** Analysis of changes in cell cycle transition between OL-N.C. and OL-miR-199a-1-5p-OE cells. The number of G1 phase (**K**, **P < 0.01) and S phase (**L**, ***P < 0.001) cells in OL-N.C. and OL-miR-199a-1-5p-OE cells.

To determine whether miR-199a-1-5p regulates CDKN1B expression in OL and POL cells, we compared its expression by RT-qPCR in OL vs POL (Fig. 5A), OL + PBS vs. OL + POL-Exo (Fig. 5B), and ON-NC and OL-miR-199a-1-5p-OE cells (Fig. 5C), respectively. CDKN1B expression was lower in POL, OL + POL-exo, and OL-miR-199a-1-5p-OE cells in which miR-199a-1-5p expression was either higher or overexpressed (Fig. 5A–C). Thus, miR-199a-1-5p regulates the expression of CDKN1B in OL and POL cells.

Tumor suppressive and anti-metastatic activities of CDKN1B are achieved via its inhibition of cell cycle progression [25–27]. As expected, in POL cells in which miR-199a-1-5p expression is higher than that of in the OL cells, there were significantly less proportion of cell in G1-phase and more in the S-phase (Fig. 5D–F). The positive correlation between miR-199a-1-5p expression and S-phase cell number, and the inverse correlation between miR-199a-1-5p expression and G1-phase cell number were also seen in OL + PBS vs. OL + POL-Exo cells (Fig. 5G–I), and in OL-NC vs. OL-miR199a-1-5p-OE cells (Fig. 5J–L). These observations indicate that POL exosomal miR-199a-1-5p, upon taking up by OL cells, can promote metastatic colonization by targeting CDKN1B which in turn releases its inhibition on cell cycle progression.

**Fig. 6:**
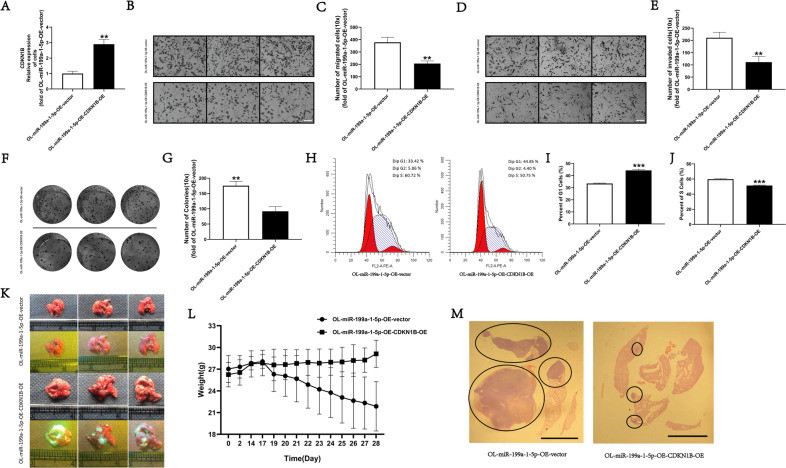
CDKN1B decrease the metastatic colonization ability of miR-199a-1-5p augmented OL in vitro and in vivo. **A** CDKN1B gene expression in OL-miR-199a-1-5p-OE-vector and OL-miR-199a-1-5p-OE-CDKN1B-OE cells evaluated by RT-qPCR. **P < 0.01. Visualization (**B**, bar = 60 μm) and quantitative analysis of (**C**, **P < 0.01**)** the migration ability between OL-miR-199a-1-5p-OE-vector and OL-miR-199a-1-5p-OE-CDKN1B-OE cells. Visualization (**D**, bar = 60 μm) and quantitative analysis of (**E**, **P < 0.01) the invasive ability between OL-miR-199a-1-5p-OE-vector and OL-miR-199a-1-5p-OE-CDKN1B-OE cells. Visualization (**F**) and quantitative analysis of (**G**, **P < 0.01) the clonogenic ability between OL-miR-199a-1-5p-OE-vector and OL-miR-199a-1-5p-OE-CDKN1B-OE cells. **H** Analysis of changes in cell cycle transition between OL-miR-199a-1-5p-OE-vector and OL-miR-199a-1-5p-OE-CDKN1B-OE cells. The number of G1 phase (**I**, ***P < 0.001) and S phase (**J**, ***P < 0.001) in OL-miR-199a-1-5p-OE-vector and OL-miR-199a-1-5p-OE-CDKN1B-OE cells. **K** Analysis of the effect of CDKN1B overexpression on metastatic colonization in vivo. **L** The weight changes of mouse injected with OL-miR-199a-1-5p-OE-vector and OL-miR-199a-1-5p-OE-CDKN1B-OE cells. **M** H&E staining of metastatic nodules in the lungs, bar = 5 mm.

To confirm that augmentation of cell cycle progression by miR-199a-1-5a is achieved through targeting CDKN1B, we performed rescue experiments. We restored CDKN1B expression in OL-miR199a-1-5p-OE cells by lentiviral mediated overexpression and obtained OL-miR199a-1-5P-OE-CDKN1B-OE cell line (Fig. 6A). Restoration of CDKN1B expression effectively prevented the observed enhancement of OL cell migration (Fig. 6B, C), invasion (Fig. 6D, E), and clonogenic colonization (Fig. 6F, G), and cell cycle progression (Fig. 6H–J) in vitro resulted from miR-199a-1-5p overexpression. In vivo, while SCID/NOD mice receiving tail vein injection of OL-miR199a-1-5p-OE-vector cells developed extensive lung metastasis (Fig. 6J), mice injected with OL-miR199a-1-5p-OE-CDKN1B-OE cells failed to develop macroscopic lung metastases (Fig. 6J) and suffered less weight loss (Fig. 6L). Restoration of CDKN1B expression also significantly reduced lung microscopic metastases by H & E analysis (Fig. 6M). Collectively, we show that the pro-metastatic effect of POL exosomal miR-199a-1-5p on OL cells is mediated the cell cycle inhibitor CDKN1B.

## Discussion

The high mortality rate of metastatic melanoma is related to the efficient metastatic colonization at the involved organs. Proliferative colonization, the rate-limiting step of metastasis [6–8], requires the formation of a new microenvironment. This process involves the interplay of a plethora of factors and mechanisms [9]. Heterogeneity also arises during metastatic progression [28–30] and displays as differences in cellular morphology, molecular features, and functions [31]. The role of the exosomes in the establishment of metastatic niche or microenvironment is at the forefront of cancer research. The exosomal crosstalk involves the transfer of “messengers” including miRNA, mRNA, and proteins between donor cells and the neighboring recipient cells thus is capable of changing the biological properties of the recipient cells [11].

In melanoma research, normal melanocytes, upon taking up exosomes secreted by melanoma cells undergo malignant transformation [32]. Exosomes secreted by melanoma cells can target surrounding normal cells such as mesenchymal stem cells [33], immune cells [34, 35], and myofibroblasts [36, 37] to make a favorable microenvironment for melanoma growth and progression. These findings show that exosomes can mediate the interactions between melanoma cancer cells and normal cells which may play an important role in melanoma oncogenesis and progression.

The present study has made the following novel findings to elucidate a new exosome-based mechanism that underlies the melanoma progression and heterogeneity:

First, exosomes mediated cancer cell-cancer cell interactions between cancer cells with different metastatic capability, affords more efficient metastatic progression. Previous studies on the role of exosomes in metastasis have been mainly focused on cancer cell-derived exosomes in modulating the functions of stromal cells [14]. Whether the extravasated neighboring cancer cells at the distant organ can alter the metastatic properties of one another has not been demonstrated prior to this report.

The lack of clinically relevant metastatic melanoma cellular and in vivo models has limited the mechanistic investigation. In this study, we employed the paired metastatic M14-POL and OL melanoma cell lines that we have developed and characterized [18]. The difference of proliferative colonization between the POL and OL cells in vivo affords us to investigate the hypothesis that “cancer cell-cancer cell interactions may promote metastatic progression and give rise to heterogeneity (i.e., multiclonal origin of metastasis)”. Exosomal profiling provides a new molecular feature that can differentiates cancer cells endowed with different metastatic capability (Fig. 2B).

Observations derived from this study have also raised new inquire, such as: can exosomes derived from low-metastatic cancer cells reduce the invasiveness of the highly metastatic cancer cells thus hinder metastatic progression? If the influence is mutual, what regulates the balance that determines the clinical outcome? Future studies are warranted.

Second, exosomal miR-199a-1-5p from highly metastatic cancer cells augments the invasiveness of low metastatic cancer cells, i.e., it is pro-metastatic. The involvement of miR-199a-1-5p in oncogenesis and progression has been reported. While it exhibits largely the tumor suppressive activity, both tumor suppressive and oncogenic functions of miR-199a-1-5p have been observed in melanoma research [21–23, 38]. The reported differences may be due to the differences in the context, the cell models, in vivo experimental design and etc. In the present study, we identified the pro-oncogenic activity of miR-199a-1-5p in the context of metastatic colonization and in our cellular models (Fig. 3). The clinical relevance of this finding was confirmed by bioinformatic analyses of the TCGA database (Fig. 2). miR-199a-1-5p expression in metastatic and recurrence tumor samples appeared significantly higher than that in the primary tumor samples (Fig. 2J). High miR-199a-1-5p expression in metastatic and recurrence patients was significantly associated with poor OS and DFS (Fig. 2K–P). Thus, miR-199a-1-5p is a poor prognostic marker for melanoma and appear pro-metastatic, consistent with our observations in the POL and OL cellular models we employed. Validation of the prognostic value of exosomal miR-199a-1-5p with clinical cohorts of melanoma is required. TCGA analysis results have validated the clinical relevance of the POL and OL models.

Third, the pro-metastatic activity of miR-199a-1-5p is achieved through targeting the cell cycle inhibitor CDKN1B. Since the hypothesis that metastatic capability can be modified by the paracrine cancer cell-cancer cell interaction via exosomal miRNA has never been tested, as a proof of principle, we choose to identify a validated mechanism to prove this hypothesis. Target Scan and KEGG pathway analysis and the literature review identified CDKNIB, a well-characterized cell cycle inhibitor and a gene target for the oncogenic activity of miR-199a-1-5p in sarcoma [24] for mechanistic validation. Although anti-oncogenic function of CDKN1B in melanoma has been reported, exosomal miR-199a-1-5p targeting of CDKN1B in the recipient cells has not been reported prior to this study. CDKN1B expression was lower in POL cells, in POL exsosomes and in OL cells co-cultured with POL cells which was negatively correlated with the corresponding miR-199a-1-5p expression (Fig. 6). Restoration of CDKN1B expression effectively prevented the observed enhancement of OL cell migration (Fig. 6B, C), invasion (Fig. 6D, E), clonogenic colonization (Fig. 6F, G), and cell cycle progression (Fig. 6H–J) in vitro, and the development of lung metastasis in vivo (Fig. 6K–M), resulted from miR-199a-1-5poverexpression. Collectively, we show that the pro-metastatic effect of POL exosomal miR-199a-1-5p on OL cells is achieved via targeting the cell cycle inhibitor CDKN1B. In depth mechanistic characterization of the miR-199a-1-5P-CDKN1B axis merits further investigation that is beyond the scope of the current study. In addition, it was reported that miR-199a-1-5p can inhibit the expression of genes involved in cancer stem cell renewal [39–41]. The expression of Aldh1, Bmi1, Klf4, and Sox2 were increased in OL cells overexpressing miR-199a-1-5p (data not shown). Thus, POL cells may transfer their highly invasive capability to OL cells by augmenting stem cell-like properties. Future studies directed at investigating the role of exosomal miR-199a-1-5p in the regulation of cancer stem cell renewal are warranted.

In conclusion, we show for the first time that exosomal crosstalk between metastatic cancer cells is a new mechanism that underlies cancer metastasis and heterogeneity. Highly metastatic melanoma cells (POL) can transfer their “metastatic power” to low-metastatic melanoma cells (OL). POL achieves this goal by utilizing its exosomes to deliver functional miRNAs, such as miR-199a-1-5p, to the targeted OL cell which in turn inactivates cell cycle inhibitor CDKN1B and augments metastatic colonization.

## Materials and methods

### Cell culture

GFP-labeled M14 were kindly provided by Dr. Robert Hoffman (University of California San Diego). M14, OL, POL, OL-N.C., OL-miR-199a-1-5p-OE, OL-miR-199a-1-5p-OE-vector, and miR-199a-1-5p-OE-CDKN1B-OE were maintained in DMEM high glucose supplemented (Hyclone) with 10% fetal bovine serum (FBS) (ExCell Bio, Shanghai, China), and incubated at 37 °C in humidified air containing 5% CO_2_.

### OL and POL cell co-culture assay

The six-well Transwell inserts (8 μm pore size, Corning) were used to separate OL and POL in the assembly of the co-culture assay. More specifically, 1 × 10^5^/well OL and POL cells were seeded into the lower and upper chamber of the Transwell respectively, and co-cultured for 48 h. For experiments in which POL cells were treated with shRAB27A to inhibit the secretion of exosomes, the co-culture assay was set up after 48 h of shRAB27A treatment.

### Transwell migration and invasion assays

Transwell inserts (8 μm pore size, BD Falcon) were used to perform invasion assay with Matrigel (100 μl, 1:8 dilution in serum-free medium, BD Biosciences) and migration assay without Matrigel. 300 μl serum-free medium containing 3 × 10^4^ cells were seeded into the upper chamber, while 800 μl medium containing 20% FBS as chemo-attractant was added into the lower chamber. After 24 h of incubation, cells were removed from the upper surface of the porous membrane with a cotton swab, followed by fixation of cells migrated and invaded to the lower surface of the membrane with 70% ethanol for 1 h and staining with crystal violet for 15 min. The stained cells were counted under light microscopy and 10 random fields from three replicate Transwells were counted. The number of migrated and invaded cells were presented as the number of cells counted per field of the porous membrane.

### Exosome isolation and identification

Exosomes were obtained by differential ultracentrifugation. After centrifugation at 800 × *g* for 5 min, the culture supernatants were collected and centrifuged at 2000 × *g* at 4 °C for 10 min. For differential ultracentrifugation, the supernatants from low-speed centrifugation were centrifuged at 10,000 × *g* at 4 °C for 30 min and then filtered through a 0.22 μm filter (EMD Millipore, Billerica) to remove the cell debris and. Afterwards, the supernatants were centrifuged at 100,000 × *g* at 4 °C for an additional 70 min, and the resultant pellets were resuspended in 100 μl PBS and stored at −80 °C for further analysis.

Protein content of the exosomes was determined by using a BCA protein 7 assay kit (Pierce, Pierce County, WA). The morphology of the exosomes was examined by transmission electron microscopy (JEM-1400PLUS, JEOL). The isolated exosomes were diluted in PBS and analyzed using a Nanoparticle Tracking Instrument (Particle Metrix) according to the manufacturer’s protocol. The sizes and concentration of exosomes were then analyzed by using the Zetaview Analysis software.

### Exosome labeling and imaging analysis

Exosomes from POL cells were labeled with PKH26 fluorescent dye (Sigma) according to the manufacturer’s instructions. For POL cells transfected with miR-N.C. mimics or miR-199a-1-5p mimics, the mimics were labeled with Cy5 fluorescent dye (Sigma) according to the manufacturer’s instructions. Dye-labeled exosomes was added to OL cells and incubated at 37 °C for 18 h. The nuclei were stained with DAPI for 15 min and washed twice with PBS before confocal microscopy (TCS SP2, LEICA) analysis. A time course (0, 6, 12, 18 h) of exosomes uptake was conducted.

### Cell colony formation assay

A total of 300 cells in 1 ml DMEM containing 0.2% agar (Solarbio Science & Technology Co., Beijing, China) and 10% FBS were plated per well into six-well plates coated with 1 ml DMEM containing 0.5% agar and 10% FBS. After 1 week, colonies were stained with crystal violet. Colonies consist of no less than 50 cells were counted. The experiment was performed in triplicates and repeated three times.

### miRNA sequencing

RNA extraction, quality testing, library construction, and sequencing were performed at BGI, Wuhan, China.

### Lentivirus-mediated stable overexpression of miR-199a-1-5p and CDKN1B in OL cells

The lentivirus particles of N.C., miR-199a-1-5p overexpression (OE), and CDKN1B overexpression (OE) plasmids were purchased from Shanghai GeneChem Company. Cells were infected with a multiplicity of infection (MOI) of 50 according to the manufacturer’s protocol. Stable OL-N.C., OL-miR-199a-1-5p-OE, and OL-miR-199a-1-5p-OE-CDKN1B-OE cells were purified by fluorescence flowcytometry sorting. After 72 h of infection, RT-qPCR and Western blotting assays were performed to confirm the infection efficiency.

### Lipofectamine 2000 transfection of miR-199a-1-5p mimics and RAB27A siRNA

RAB27A siRNA, miR-199a-1-5p mimics and N.C control mimics were obtained from GenePharma. Cells were transfected with the siRNA or miRNA mimics by Lipofectamine 2000 according to the manufacturer’s instructions. After 72 h of transfection, RT-qPCR and western blotting assays were performed to measure the silencing or overexpression efficiency.

### Real-time quantitative polymerase chain reaction (RT-qPCR)

Total cellular RNA was extracted with Trizol (TaKaRa, Dalian, China). Two micrograms of total RNA was subjected to reverse transcription with PrimeScript RT Master Mix (TaKaRa, Dalian, China). For the reverse transcription of miRNA, Mir-X miRNA First-Strand Synthesis Kit (TaKaRa, Dalian, China) was used. RT-qPCR was performed using a SYBR Green Real-time PCR Master Mix kit (TaKaRa, Dalian, China) under the following condition: initial pre-incubation at 95 °C for 30 s, followed by 39 cycles at 95 °C for 5 s and 60 °C for 30 s. The relative mRNA levels were analyzed using the 2−ΔΔCt method. The forward primer sequence for miR-199a-1-5p was GCCCAGTGTTCAGACTACCTGTTC, and for U6 was GCTTCGGCAGCACACATACTAAAAT. The forward primer sequence and reverse primer sequence of RAB27A were TCTGGTGTAGGGAAGACCAGT and TACGAAACCTCTCCTGCCCT, respectively. The forward primer sequence and reverse primer sequence of CDKN1B were GCAAGTACGAGTGGCAAGAGGTG and CCGCTGACATCCTGGCTCTCC, respectively. The forward primer sequence and reverse primer sequence of GAPDH were AGAAGGCTGGGGCTCATTTG and AGGGGCCATCCACAGTCTTC, respectively.

### Western blotting analysis

Cells were lysed in 200 μl SDS lysis buffer (Beyotime, Shanghai, China) with 1% PMSF (Beyotime, Shanghai, China) and boiled at 100 °C for 15 min. Forty micrograms of each protein sample was separated by electrophoresis with 12% polyacrylamide gels and transferred to polyvinylidene fluoride (PVDF) membranes (Bio-Rad, CA, USA). Thereafter, according to the manufacturer’s instructions, the proteins were incubated with appropriate primary antibodies and secondary antibodies. Primary antibodies of Alix, CD63, CD81, Calnexin, RAB27A, and the loading control (GAPDH) were purchased from Proteintech Group. Results were analyzed by ImageJ version 1.47 (National Institutes of Health, Maryland, USA). Three independent experiments were done for statistical analysis.

### Cell cycle analysis

Cells were fixed with ice-cold isopropanol overnight. Cell cycle analysis was conducted by Flowcytometry (FACSVantage SE, BD). DNA content was analyzed by ModFit Lt.

### Animal experiments

All mice used in the study were obtained from the core facility of the Experimental Animal Centre in Chongqing Medical University. All animal work was conducted in accordance with an approved protocol and carried out in accordance with the institutional animal welfare guidelines of the Chongqing Medical University. 10^6^ tumor cells were transplanted into each male NOD/SCID mouse via tail intravenous injection. Mice were weighed every 3 days and dissected after 35 days post tumor cell injection. Sellstrom Z87 fluorescence goggles and an LDP 470 nm bright blue flashlight were used for examining the metastases in the mice. Metastatic nodules in the lungs were counted at the time of sacrifice and confirmed by H&E staining.

### Bioinformatics analysis

TargetScan 7.2 (http://www.targetscan.org/vert_72/) was used for identifying candidate gene targets of the miRNA of interest. miR-199a-1-5p expression and mRNA expression of CDKN1B in human melanoma was analyzed by TCGA (http://cancergenome.nih.gov), ASSISTANT for Clinical Bioinformatics (https://www.aclbi.com/static/index.html#/), GEPIA2 (http://gepia2.cancer-pku.cn/#index). To analyze the survival of melanoma, patient samples were also analyzed by TCGA database, ACLBI database, GEPIA2 database and OncoLnc database (http://www.oncolnc.org/). TIMER (https://cistrome.shinyapps.io/timer/) was used to analyze the association of CDKN1B and immune response.

### Statistical analysis

All experiments were performed at least three times for statistical analysis. Quantitative results were shown as mean ± SEM (standard error of the mean). Data were analyzed with GraphPad Prism version 5.0 (GraphPad Software Inc., CA, USA) by two-tailed Student’s *t*-test. Images were globally adjusted with Photoshop version 11.0.1 (Adobe Systems Inc., CA, USA). *P < 0.05 was considered significant statistically and was marked with an asterisk. **P < 0.01 and ***P < 0.001 were considered highly statistically significant and were marked with double asterisks and triple asterisks, respectively.

## Supplementary information

Original Data File

## Data Availability

The data used to support the findings of this study are available from every author upon request.
