# Supplementary figures and images for: Low-metastatic melanoma cells acquire enhanced metastatic capability via exosomal transfer of miR-199a-1-5p from highly metastatic melanoma cells
Source: Cell Death Discov. 2022 Apr 9;8:188. doi: 10.1038/s41420-022-00993-8 (PMC8994777; doi:10.1038/s41420-022-00993-8)

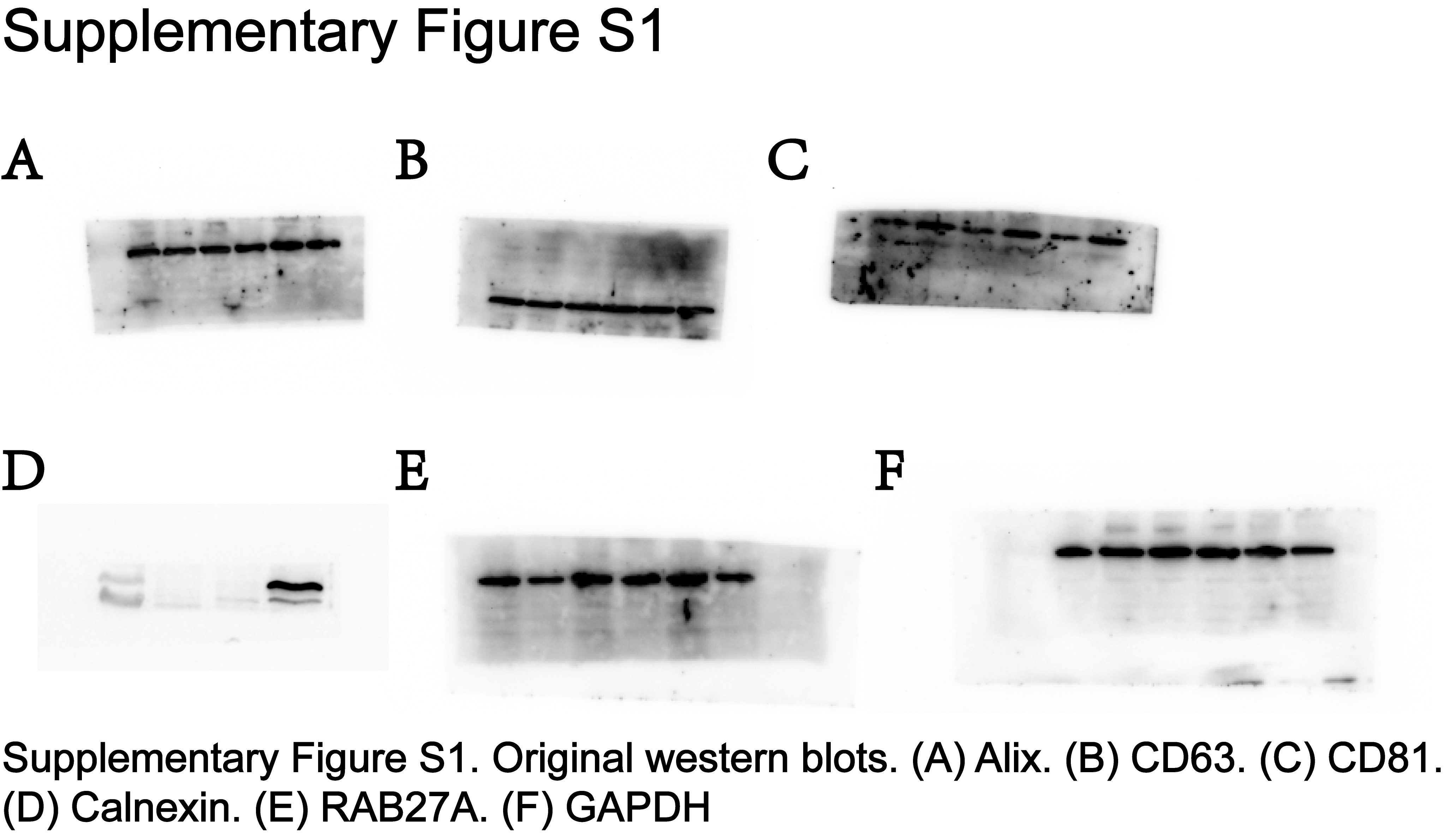

Supplement: Supplementary file 1 — Original Data File [file 41420_2022_993_MOESM1_ESM.tif]
